# Influence of surface peripheral electrical stimulation on nerve regeneration after digital nerve neurorrhaphy: study protocol for a randomized clinical trial

**DOI:** 10.12688/f1000research.42120.2

**Published:** 2021-12-06

**Authors:** Enilton Mattos, Alex Guedes, Paulo Itamar Ferraz Lessa, Abrahão Fontes Baptista

**Affiliations:** 1 Pos Graduate Program in Medicine and Human Health, Escola Bahiana de Medicina e Saúde Pública, Salvador, Bahia, Brazil; 2 Professor Edgard Santos University Hospital Complex, Salvador, Bahia, Brazil; 3 Bahia Medical School, Federal University of Bahia, Salvador, Bahia, Brazil; 4 Bahia State University, Salvador, Bahia, Brazil; 5 Center for Mathematics, Computation and Cognition, Federal University of ABC, São Bernardo do Campo, São Paulo, Brazil; 6 Laboratory of Medical Investigations 54 (LIM-54), São Paulo University, São Paulo, São Paulo, Brazil

**Keywords:** Transcutaneous Electric Nerve Stimulation, Peripheral Nerves, Nerve Regeneration, Electric Stimulation Therapy, Peripheral Nerve Injury

## Abstract

We will study the influence of low intensity and frequency surface peripheral electrical stimulation (PES) on nerve regeneration of digital nerve injuries of the hand after its surgical repair in humans. Participants will be patients with acute traumatic peripheral nerve injury referred to the Hand Surgery Service of the General Hospital of the State of Bahia, a reference service in the state. These patients will undergo surgery followed by PES in the immediate postoperative period. After hospital discharge, they will be followed up on an outpatient basis by researchers, who will remotely supervise a physiotherapy program. Our hypothesis is that PES will positively influence the recovery of sensory function in patients undergoing neurorrhaphy of digital nerves of the hand.

**ReBEC registration:**  U1111-1259-1998 (12/18/2020)

## Introduction

### Peripheral nerve injury and regeneration

Damage to the peripheral nervous system (axonal damage) can occur due to a variety of reasons including trauma, ischemia, or inflammation ( Stoll & Müller, 1999). In response to peripheral nerve injury, morphological and functional changes can occur ( Knight, 2000; Robbins, 1974), such as motor and sensory function impairment, hyperesthesia, and low-temperature intolerance ( Rosén & Lundborg, 2000). The process of extensive degeneration that occurs when the axon distal to the injury degenerates is known as Wallerian degeneration, however, the proximal stump, which remains attached to the cell body, can regenerate, and grow towards the target organ ( Stoll & Müller, 1999).

Neural regeneration following injury is influenced by a number of factors, such as age, level and extent of the injury, time elapsed from injury to repair, and presence of associated injuries ( Dunlop *et al.*, 2019; Ruijs *et al.*, 2005). Axonal regeneration is influenced by chemotactic and electrical tracks ( Song *et al.*, 2004; Stoll & Müller, 1999): if the tracks are not adequate (high or long), or the distance to be covered by the axonal sprouts is too large, the regenerated neurons will not be functional. This is the case of proximal injuries occurring close to the cell bodies, such as traumatic injury to the brachial plexus and injury of nerve roots in the conjugation foramen ( Gordon *et al.*, 2003). When the metabolism or nutrient supply is affected, such as in diabetes, functional regeneration is also impaired ( Kennedy & Zochodne, 2005). Therefore, promoting functional regeneration is key for the peripheral nervous system to resume normal functions.

Strategies to improve peripheral nerve regeneration include the use of neurotrophic factors, stem cells ( Lopes *et al.*, 2006; Tohill & Terenghi, 2004), ultrasound ( Crisci & Ferreira, 2002), low-intensity LASER ( Bae *et al.*, 2004), physical exercise ( Molteni *et al.*, 2004; Seo *et al.*, 2006), and electromagnetic fields. The latter is based on the fact that tissue injury results in an influx of calcium that causes endogenous electrical currents by increasing local electrical potentials ( McCaig *et al.*, 2002; Watson, 1998). These currents are formed through electrical gradients between the affected area and the surrounding regions and remain active throughout the regenerative process ( Low & Reed, 2001). Exogenous electrical stimulation has been used to promote neural regeneration and early tissue recovery following injury.

### Electric fields and peripheral nerve regeneration

Electrical stimulation to improve the rate and speed of peripheral nerve regeneration involves the application of electrical fields of constant or varying frequency, as demonstrated in animal studies ( Baptista *et al.*, 2007, 2008). Electrical currents are usually used, either flowing unidirectional (monophasic, constant or pulsed electrical currents) or bidirectional (alternate or biphasic electrical currents). The electrical currents may be administered through electrodes implanted in the nerve itself, intraoperatively, and using percutaneous or transcutaneous stimulation. Monophasic electrical currents have the advantage of unidirectionality; they can generate electrophoretic effects on membrane proteins and, thus, guide neurite growth towards the cathode ( McCaig *et al.*, 2002). However, if used at high intensity or for long periods, the monophasic electrical currents can cause harmful effects due to electrophoresis itself ( Low & Reed, 2001). Although biphasic currents do not offer the risk of harmful electrophoretic effects, they are less commonly used, as they do not have the power to guide the neurites towards the target organ.

There are different types of electrodes, with some predominating over others. In general, the closer the electrode is to the nerve, the smaller the current amplitude, as there is no need to overcome the impedance exerted by the surrounding tissues.

***Implanted electrodes.*** Stimulation with implanted electrodes and different types of electrical currents have been the most used method since the 1980s in experimental models, generally for lesions in the soleus ( Nix & Hopf, 1983) and sciatic ( Beveridge & Politis, 1988; Kerns *et al.*, 1991; Mendonça *et al.*, 2003; Politis *et al.*, 1988; Pomeranz & Campbell, 1993) nerves. All of these studies showed positive results, with improvements in functional and/or morphological parameters.

Only two studies—one on lesions of the common fibular nerve ( McGinnis & Murphy, 1992) and the other on lesions of the sciatic nerve ( Hanson & McGinnis, 1994)—reported negative results, with fewer regenerating fibers, formation of neuromas, and a lower rate of myelination in the stimulated groups. However, in these studies, one of the electrodes was placed inside a small silicone tube that joined the two sectioned stumps, leading to the interaction of the single-phase electric current with the material of the tube. Deleterious effects arose due to the lack of alternating current and, consequently, destruction of part of the tube, with physical impediment to the passage of neurites.

To avoid tissue destruction, although the electrical currents used in all of the above-mentioned studies were monophasic, electrical current amplitudes were extremely low (below the sensitive threshold in the μA range). Stimulation was usually performed continuously for a few weeks prior to analysis.

***Percutaneous electrostimulation.*** Percutaneous electrodes are usually acupuncture needles inserted into the skin and connected to an electric current generator. Electric currents through percutaneous electrodes have been used in experimental models and perhaps they are, of the methods studied so far, the simplest to apply in clinical practice. This type of stimulation has been used in models of sciatic nerve injury ( Chen *et al.*, 2001; Inoue *et al.*, 2003; McDevitt *et al.*, 1987; Pomeranz *et al.*, 1984), with the best results achieved when the cathode was placed distal and the anode proximal to the lesion. Monophasic electrical currents were used, with amplitudes below the sensory threshold, yet reaching the intensity of 1 mA (much higher than that used in techniques with implanted electrodes).

***Intraoperative electrostimulation.*** In intraoperative electrostimulation, the nerve is stimulated shortly following injury, for varying periods of time. Studies on intraoperative electrostimulation have mostly used the sciatic ( Scott, 1991) and femoral ( Al-Majed *et al.*, 2000a, 2000b, 2004; Brushart *et al.*, 2002) nerves. The first studies used monophasic electrical currents; the most recent ones used alternating currents with pulse frequencies of 20 Hz ( Al-Majed *et al.*, 2000a, 2000b, 2004; Brushart *et al.*, 2002). The latter allowed more precise and faster reinnervation; increased the expression of brain-derived neurotrophic factor (BDNF), tyrosine receptor kinase B (TrKB), Tα1-tubulin, and growth associated protein 43 (GAP-43), and other genes/proteins associated with regeneration; and reduced expression of neurofilaments (a phenomenon associated with regeneration). This model has been the only one to be translated to humans, so far. Gordon *et al.* (2010) evaluated the same electrical stimulation protocol in patients with denervation of the muscles of the thenar region due to carpal tunnel syndrome. They observed that the treatment accelerated axonal regeneration without affecting function. Intraoperative electrostimulation differs from others, in addition to the form of stimulation, for the brief time it is used (approximately 1–2 hours of stimulation following injury).

***Thin-film wireless implantable nerve stimulators.*** Using widely established technology, wireless devices dedicated to peripheral nerve stimulation have been used to improve functional recovery in a rodent model, without the additional time needed to provide direct stimulation during the surgical procedure ( Ray *et al.*, 2017). This strategy has not yet had practical application in humans.

***Surface electrodes.*** The use of transcutaneous surface electrodes is a non-invasive option that can be used for a longer period, especially when associated with biphasic electrical currents. Its handling is practical and simple, avoiding the reactions provoked by implant surgery or percutaneous stimulation.

Previous studies have evaluated the influence of peripheral electrical stimulation (PES) through surface electrodes on the regeneration of tissues such as tendons ( Burssens *et al.*, 2003, 2005), skin ( Kaada & Emru, 1988; Kjartansson *et al.*, 1988; Khalil & Merhi, 2000; Liebano *et al.*, 2003), and bone ( Kahn, 1982), with varying results. This modality is associated with effects such as increased blood flow (from Sandberg *et al.*, 2007; de Vries *et al.*, 2007) and collagen synthesis ( Burssens *et al.*, 2005). However, electrical currents with amplitudes above 1 mA are needed, which may be related to decreased concentrations of adenosine triphosphate (ATP) ( Cheng *et al.*, 1982) and, consequently, inhibition of peripheral nerve regeneration.

Electrical fields may influence blood circulation/perfusion, promoting an increase in axonal sprouting and nerve regeneration ( McCaig *et al.*, 2005). Low frequency electrical currents can selectively activate sensory C fibers and increase the expression of neuropeptides such as substance P, which generate vasodilation ( Burssens *et al.*, 2005; Kashiba & Ueda, 1991; Kjartansson *et al.*, 1988). Also, low-frequency PES (2–10 Hz) is usually associated with rhythmic muscle contraction, which can have circulatory effects ( Dobsák *et al.*, 2006). However, recent evidence has suggested that high-frequency PES, which is used without producing muscle contraction, also increases circulation. de Vries *et al.* (2007) demonstrated an increase in coronary circulation as a result of high-frequency PES.

The most studied effects of PES are related to pain control. Analgesia caused by high-frequency PES activates δ-opioid receptors, while low-frequency PES activates μ-opioid receptors ( Kalra *et al.*, 2001; Sluka & Walsh, 2003). Sinatra & Ford (1979) demonstrated that the chronic use of morphine for 14 days leads to a delay in peripheral nerve regeneration, as reflected by a lower number of axonal profiles, decreased removal of myelin debris, and hypertrophy and proliferation of Schwann cells. Zeng *et al.* (2007) also demonstrated that exposure to morphine, acting via μ-opioid receptors, increases the regeneration of unmyelinated fibers, while inhibiting the regeneration of myelinated fibers after ischiatic nerve crush injury.

The fundamental difference between the studies that showed stimulating or inhibitory effects on the regeneration of the peripheral nervous system via the activation of opioid receptors was the prolonged time of use, which may have led to the development of pharmacological tolerance. Chandran & Sluka (2003) demonstrated that repeated high- and low-frequency PES for 20 minutes a day, led to opioid tolerance on day 4. Mao *et al.* (2002) demonstrated that this effect is mediated by the *N*-methyl-D-aspartate (NMDA)–caspase pathway, which leads to apoptosis of neuronal cells in the spinal cord. Therefore, the prolonged use of PES can lead to the development of opioid tolerance and neurotoxicity of the cells involved in regeneration. A brief use of PES, which does not lead to opioid tolerance, could however, be an important strategy to promote regeneration ( de Assis *et al.*, 2014), but this has not so far been investigated in humans.

### Objectives

Our overall aim is to study the influence of PES on peripheral nerve regeneration in humans. Specifically, we will study the influence of PES on recovery of sensory function, and on the social participation of patients undergoing neurorrhaphy of digital nerves of the hand.

### Protocol

This is a randomized, double-blinded, controlled clinical trial that will be carried out in a reference tertiary general hospital in Bahia, Brazil. This protocol has been registered at ReBEC (U1111-1259-1998 on 18 ^th^ December 2020) and follows the Standard Protocol Items: Recommendations for Interventional Trials (SPIRIT) checklist ( de Santana Ribeiro de Mattos *et al.*, 2021d). Patients undergoing neurorrhaphy for injuries caused by transection of digital nerves in the hand will be involved in the study. Half the participants will have PES (Endophasys, KLD, Brazil) performed immediately after surgery and before hospital discharge and the other half will receive a SHAM treatment. The participants and outcome assessors will be blinded to patient allocation. The electrodes will be placed in the same position and for the same amount of time for the intervention group as the sham group. The outcome measures evaluators will not know which intervention was assigned to the participants.

### Participants

A convenience sample of adult patients (over 18 years old of both sexes) with acute traumatic non-segmental digital nerve injury referred from others health centers to the General Hospital of the State of Bahia to undergo surgical repair within a maximum of 2 weeks after the injury will be included. Patients with metal implants at the surgery site, who have a history of seizures, who have a cardiac pacemaker, or who have a local infection or skin lesion that prevents the application of surface electrodes will be excluded from the study.

### Materials

Basic material: Endophasys Electrostimulator – KLD, Brazil and silicone-carbon electrodes (used to perform electrostimulation in the patients).

All materials required for this study are available as extended data ( de Santana Ribeiro de Mattos *et al.*, 2021a).

### Setting

The Hand Surgery Service of the General Hospital of the State of Bahia is the foremost public tertiary trauma hospital and act mainly as referral center for other institutions in Bahia, Brazil. Outpatient Federal University of Bahia Physiotherapy Department will support the patients after surgery. The available facilities at these centers are listed in the extended data ( de Santana Ribeiro de Mattos *et al.*, 2021b).

### Intervention

**Figure 1.**
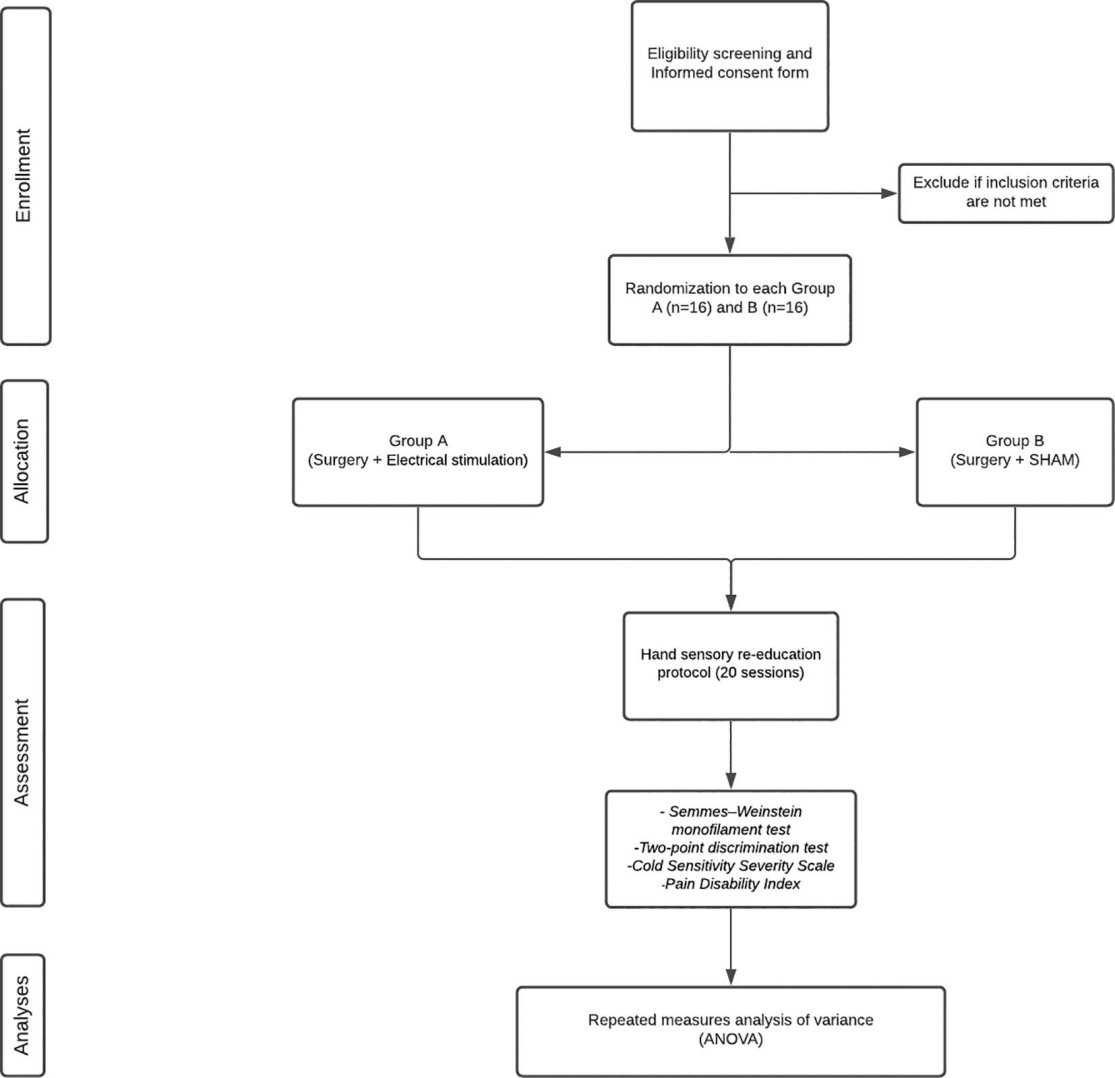
Flow chart of the study based on CONSORT criteria.

Figure 1 shows a flow diagram of the participants’ journey through the study. Patients will be divided and allocated to two groups: group A (surgery + PES); or group B (surgery + sham). A simple randomization strategy will be performed to achieve balanced groups, using an online resource ( randomization.com). The hidden allocation and electrical stimulation will be performed by a professional not involved in other stages of the study. Opaque and sealed envelopes will be used to address participant allocation. Participants will only be randomized if they provide informed consent to participate in the trial.

Following surgery, both groups will additionally receive 1 hour of either electrical stimulation (group A) or sham (group B). In total, two silicone-carbon and gel electrodes (measuring 1 × 1 cm) will be used for all participants, with one placed exactly proximal and the other exactly distal to the surgical site. The two groups will receive the following therapeutic regimens:

• *Group A: Surgery + PES.* The electrodes will produce a square-pulsed, biphasic, symmetrical current at a frequency of 20 Hz, pulse width of 0.4 ms, and amplitude at the motor threshold of the median nerve, for 1 hour directly after surgery (n = 16);
• *Group B: Surgery + sham PES.* The electrodes will produce a square-pulsed, biphasic, symmetric electric current at a frequency of 20 Hz, pulse width of 0.4 ms, and amplitude at the motor threshold of the median nerve, for 1 hour directly after surgery (the same as Group A) but the electrostimulator will be turned on only until the patient is aware of it, after which the amplitude will be set to zero (n = 16).

In the first post-operative review, the patient will be evaluated by the doctor at the outpatient clinic. A physiotherapist specialized in the area and blinded to the intervention will guide on the rehabilitation protocol that will be monitored remotely by electronic means available to the patient (WhatsApp, Skype, etc.). Patients will undergo a hand sensory re-education protocol based on the one proposed by Dellon & Jabaley (1982) for a period of 3 months. This protocol involves exercises for discrimination of static and dynamic touch and objects of different shapes, sizes, and textures guided by the physiotherapist, and the patient is also stimulated to perform a home program exercises. This will involve a total of 20 remote monitored sessions lasting about 30 minutes each, carried out, on average, twice per week. No videos or photos of the patients will be acquired to guarantee the patient’s privacy and data confidentiality.

Patients will be evaluated in person after every 10 treatment sessions by the main author of the study, who is responsible for the surgical and postoperative follow-up of the study participants but blinded to group allocation.

Participants must complete a total of 4 face-to-face assessments to document the results:

‐ 1 pre-intervention/electrical stimulation
‐ 1-week post-electrical stimulation
‐ 1 after 10 rehabilitation sessions
‐ 1 after all 20 rehabilitations sessions

The research will be completed within 1 year.

### Outcome measures

• **Primary outcome:** Improvement of peripheral nerve regeneration of digital nerves in the hand measured using quantitative sensory tests (Semmes-Weinstein monofilaments and two-point discrimination tests measured and compared in the four presential assessments). The difference between the two treatment arms (group A × group B) measured through the sensory tests (SWM and 2PD) will be evaluated after randomization.
• **Secondary outcome:** Improvement of sensory functions and social participation of the individual submitted to neurorrhaphy of digital nerves of the hand measured through questionnaires (Cold Sensitivity Severity Scale described by McCabe *et al.* (1991), and the Pain Disability Index questionnaire recommended by Novak *et al.*, 2010).

***Semmes–Weinstein monofilament test (SWM).*** The Semmes–Weinstein monofilament test is used to assess the perception of pressure thresholds, which reflect the reinnervation of peripheral targets. Using scored probes, the assessment of perception of touch/pressure is measured and recorded. This test is a prime marker of functional recovery and provides quantitative data that can be used to monitor the patient during the course of nerve regeneration ( Wang *et al.*, 2013).

Subjects were asked to place their hands over a table, and keep their eyes closed during this test. Each filament, starting with the smallest caliber, was tested over the pulp side of the affected finger. The filament was applied perpendicularly for 1 to 1.5 s in three trials. A positive response in at least 2 of the 3 trials marked the sensory threshold ( Gordon *et al.*, 2010). We used the test results for data analysis.

***Two-point discrimination test (2PD).*** The two-point discrimination test, first described by WEBER in 1835 ( Dunlop *et al.*, 2019), is an established assessment tool for tactile gnosis ( Wang *et al.*, 2013). It is defined as the distance between compass points necessary for the patient to feel two contacts ( Rosén & Lundborg, 2000). Ideally, it is recorded as an absolute value compared to the corresponding portion of the contralateral, uninjured finger. Normal values in an undamaged strip of the finger vary from 2 to 6 mm ( Table 1).

**Table 1.** Interpretation scale of the two-point discrimination test (reproduced from Silva *et al.*, 2014).

Measurement	Interpretation
2 mm to 5mm	Normal
6 mm to 10 mm	Fair
11 mm to 15 mm	Poor
One point of perception	Protective
No point perceived	Anesthesia

Classification schemes for the two-point discrimination test such as the Medical Research Council (1954), modified by MACKINNON & DELLON (Modified Highet Classification), allow to divide into groups of value ranges according to the sensitive threshold of recovery ( Table 2).

**Table 2.** Modified HIGHET’s classification (reproduced from Dunlop *et al.*, 2019).

Sensory recovery	Highet	s2PD	m2PD	Recovery of sensibility
Failure	S0			No recovery of sensibility in the autonomous zone of the nerve
Poor	S1 S1+ S2 S2+ S3	>15 mm	>7 mm	Recovery of deep cutaneous pain sensibility Recovery of superficial pain and some touch sensibility Recovery of superficial pain sensibility As with S2, but with over response Recovery of pain and touch sensibility with no over response
Good	S3+	7–15 mm	4–7 mm	As in S3, but with good localisation of the stimulus but imperfect recovery of 2PD
Excellent	S4	2–6 mm	2–3 mm	Complete sensory recovery

s2PD = static two-point discrimination; m2PD = moving two-point discrimination.

***Cold Sensitivity Severity Scale (CSS).*** The development of cold sensitivity is quite common after surgery and hand injury. After certain types of injuries, such as amputation and nerve damage, hypersensitivity can cause severe disability. The Cold Sensitivity Severity Scale developed by McCabe *et al.* (1991) provides a reliable scale to measure cold sensitivity. The CSS scale includes four questions about events in the home that cause cold-related symptoms. To respond to each item, the patient is asked to place an X on a 100 mm line reflecting the severity of the symptom. The line had indicators at 25 mm intervals and underlying descriptors. The score for each item was then measured in millimeters from the beginning of the line, with the sum of the appropriate four-item subscale giving the cold-sensitivity severity score.

**Figure 2.**
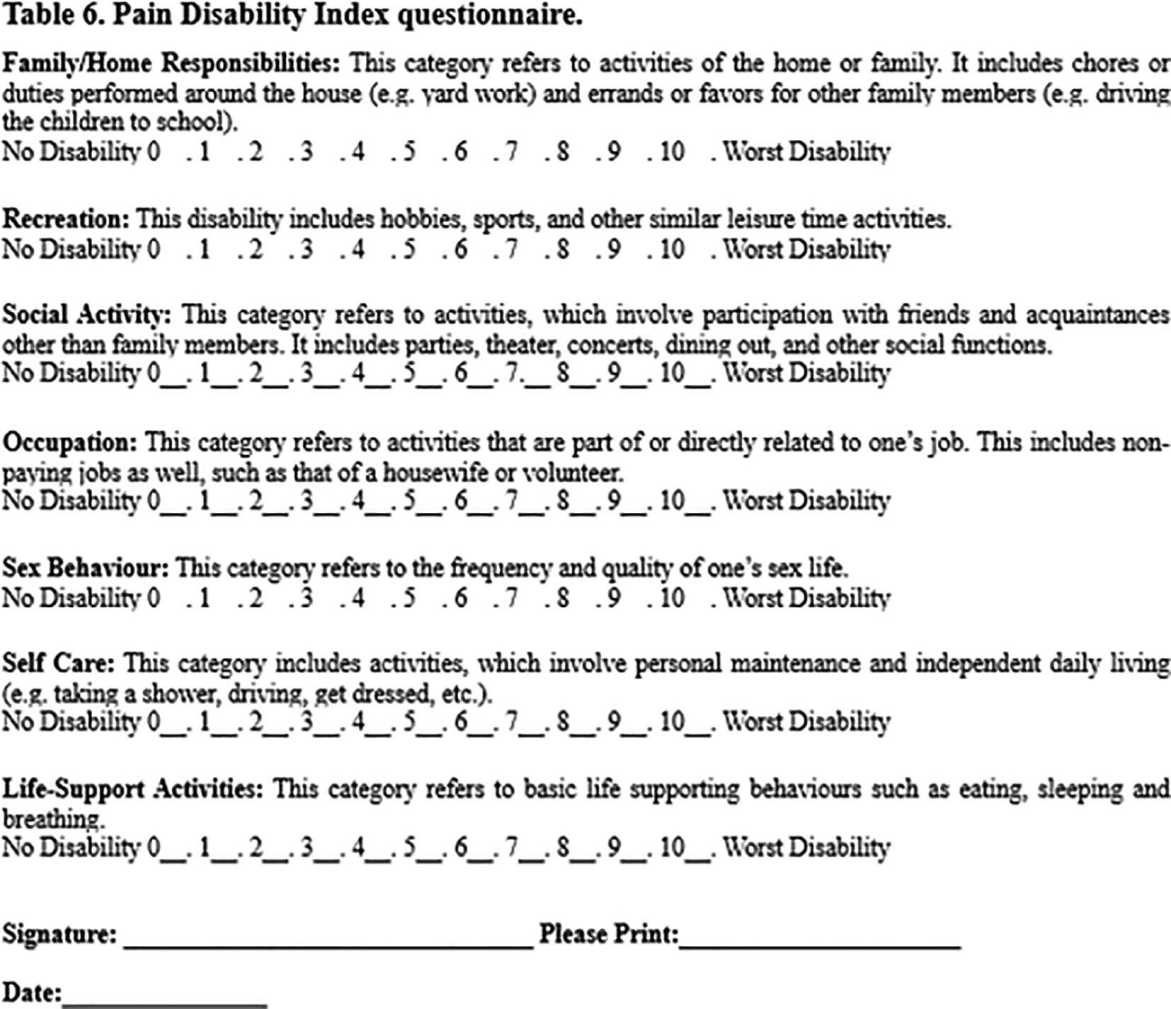
Pain Disability Index questionnaire. (Reproduced from Wang *et al.*, 2013).

***Pain Disability Index.*** The Pain Disability Index is a seven-item questionnaire designed to assess the extent to which pain interferes in the domains of daily life (family and household responsibilities, recreation, social activity, occupation, sexual behavior, self-care, and life support activity). Each item is rated on a scale from 0 (no disability) to 10 (total disability). The final score (ranging from 0 to 70) is calculated by adding the scores of each item, with a higher score indicating a higher level of disability due to pain ( Figure 2). The consistency, validity, and reliability of this questionnaire has been tested and proved to be useful in studies of nerve damage ( Novak *et al.*, 2010).

### Sample size and patient allocation

Patient allocation and statistical analysis will be done by a researcher (AFB) who will be blinded to the procedures and groups. A simple randomization will be performed using sealed and opaque envelopes. There will be 16 patients per group (groups A and B), totaling 32 patients. The sample size is based on the study by Gordon *et al.* (2010), as this is the only randomized clinical trial in the literature. We estimated the sample considering a repeated measures analysis of variance (ANOVA) test with interaction between and inter factors, with an effect size of 0.26 (according to Gordon *et al.*, 2010), alpha-type error of 5%, power of 80%, two groups, three measures, with correction between factors of repeated measures of 0.5, and correction of non-sphericity of 1. These parameters resulted in 26 individuals in the total sample (13 per group), which were increased by 20% to compensate for possible losses, resulting in a final sample of 32 patients (16 per group). The selection of the sample to compose the two groups will be carried out by an experienced (>10 years) hand surgeon, through the clinical examination and application of evaluation tools (Semmes-Weinstein monofilaments, two-point discrimination tests, Cold Sensitivity Severity Scale and the Pain Disability Index).

### Data management

The collected data will be recorded in a hand-registered logbook and typed in EXCEL, archived in Google Drive. The Cold Sensitivity Severity Scale and Pain Disability Index questionnaires are applied to each participant in the last evaluation by the hand surgeon to be answered individually and, at the end of the collection, are imported in excel format and archived in Google Drive. All data will be entered manually and electronically and stored on a password-protected laptop, computer hard drive, and external storage device in possession of the responsible for search and virtually shared with the supervisor researcher. Participant files will be stored in numerical order and stored in a secure and accessible place and manner. Participant files will be maintained in storage for a period of 5 years after completion of the study.

### Statistical analysis

All statistical tests will be performed using SPSS (V25.0, IBM Corporation, New York, USA). The data will be evaluated in a paired and non-paired way. For paired evaluations (intra-group comparisons), repeated measures ANOVA or the Friedman test will be used, followed by the Student–Newman–Keuls post-test. For non-paired assessments (comparisons between groups), analysis of variance of a measure (one-way ANOVA or the Kruskal–Wallis test) will be used, followed by the Student–Newman–Keuls post-test. The choice of tests will depend on the normality or the nature of the data.

For statistical analysis, the 95% confidence interval will be considered, with an alpha of 5% (P < 0.05) and power of 80%. Descriptive analysis will be done through the averages or medians associated with the applicable dispersion measures (standard deviation or quartiles 25/75). Both the measurements of the variables in each study and the statistical analysis will be performed blindly.

The independent variable for both groups will be the use of electric currents. The dependent variables will be derived from the pre- and post-treatment assessments (Semmes-Weinstein monofilaments, two-point discrimination, Cold Sensitivity Severity Scale and Pain Disability Index).

### Ethical aspects

Patients will be informed orally at the time of admission to the hospital about the nature of the study (including the description of the procedures that will be used, possible discomforts and risks and expected benefits, existing alternative treatment methods, monitoring and assistance approaches, and responsible staff) using accessible language.

Patients will receive clarifications before and during the study regarding the methodology, stating that we will not use a placebo group, and inclusion in a control group is possible. They will be informed about their freedom to refuse to participate or withdraw from the study, at any stage of the research, without any penalty nor prejudice to care; the confidentiality of their data; the absence of reimbursement of expenses resulting from the participation of the research; and the forms of indemnity in the event of any damages resulting from the research in accordance with the ethical standards and regulations of human studies of the Helsinki declaration (2014).

The procedures that we will use do not generate vital risks. The risks are minimal for patients, since we will use low-intensity biphasic electrical currents, which do not generate electrolytic effects on the skin, in addition to surface electrodes. The stimulation will be done only once, thus minimizing the possibility of skin irritation. None of the assessment procedures creates risks for skin injuries. If there are any complications regarding the procedures, patients will be immediately referred to the doctors who accompany them and are part of the project.

The project was submitted to the Research Ethics Committee of the Faculty of Medicine of Bahia, Federal University of Bahia. Patients who agree to participate in the study will sign an informed consent form on admission (extended data), according to Resolution No. 466 of December 12, 2012, of the National Health Council.

The information provided by the patients, as well as all data collected from the research will be kept confidential, to preserve their identity. Data will be kept in a file with a key from the Research Group on Musculoskeletal Dynamics at the Federal University of Bahia (UFBA), under the responsibility of Prof. Abrahão Fontes Baptista.

### Plans for dissemination

The study findings will be published in a thesis and research article. This work is linked to the master’s degree of Enilton de Santana Ribeiro de Mattos (Graduate Program of the Bahiana School of Medicine and Public Health, under the guidance of Prof. Abrahão Fontes Baptista and co-supervision of Prof. Alex Guedes).

### Study status

The study will start after the project has been approved by the Research Ethics Committee (CEP) of the Faculty of Medicine of the Federal University of Bahia, Federal University of Bahia and upon receiving all the materials needed. The study is scheduled to run for 10–12 months from October 2020 to October 2021 as described in Table 3 (we are at the Treatment Stage according to the Milestone schedule as of February 2020).

**Table 3.** Milestone schedule.

Milestone	Bimonthly periods				
	1	2	3	4	5
Literature review	x	x	x	x	x
Project submission to the ethics committee		x			
Treatment stage (surgical stage, electrostimulation, rehabilitation)			x	x	x
Analysis and writing of clinical results			x	x	x
Analysis and writing of statistical results				x	x
Project qualification			x		
Thesis writing			x	x	x
Submission of articles		x	x	x	x
Thesis defense and submission of the final article				x	x

## Discussion

The main contributions of this study are discussed below.

### Scientific impact

The use of electric currents to promote peripheral nerve regeneration has been studied in experimental animal models, and, essentially, through invasive techniques. Surface electrodes are cheaper and offer minimal potential risk for the patient. Despite these advantages, in general, studies with humans address the use of electrical currents to generate contraction of the denervated muscle, but not to directly promote regeneration. This study could provide novel, useful, and practical strategies to treat patients with peripheral nerve damage.

### Technological impact

We are involved with a project that has already been approved by the PAPPE Public Notice of the State of Bahia Foundation for Research Support, for the creation of functional electrical stimulation devices for patients with peripheral nerve damage. For this collaboration between the Polytechnic School and the Institute of Health Sciences, both from UFBA, some data would be important for the manufacture of these devices. It is necessary to know whether electrical currents, as they are being tested in this project, may or may not influence peripheral nerve regeneration, so that we can complete the planning of the functional electrical stimulation device.

### Economic and social impact

Peripheral nerve injuries are clinical entities that are usually underdiagnosed and undertreated. This has consequences, such as worker incapacity, with repercussions on their families and means of work. Transection injury treatments are usually exclusively surgical. However, strategies to speed recovery and return to function are extremely important both for the patients to have a greater chance of returning to work and to minimize costs related to associated complications.

In the city of Salvador, patients with peripheral nerve damage who have low purchasing power are usually operated on at the general hospital of the state and tend to have difficulties in carrying out their rehabilitation, as they do not have a service available for this situation. This research project is part of a larger perspective, with the creation of a reference service for the rehabilitation of patients with peripheral nerve damage, which should work within the Physiotherapy Service of Professor Edgard Santos University Hospital Complex, Federal University of Bahia. In addition to patients with digital nerve injuries, this service can serve all other patients who are operated on in acute situations or who have chronic neuropathies and need specialized care.

## Data availability

### Underlying data

No data are associated with this article.

### Extended data

Figshare:

List of study materials: https://doi.org/10.6084/m9.figshare.13636685 ( de Santana Ribeiro de Mattos *et al.*, 2021a)

Available facilities: https://doi.org/10.6084/m9.figshare.13636694 ( de Santana Ribeiro de Mattos *et al.*, 2021b)

Participant information sheet and consent form: https://doi.org/10.6084/m9.figshare.13681825 ( de Santana Ribeiro de Mattos *et al.*, 2021c)

### Reporting guidelines

Figshare: SPIRIT checklist for ‘Influence of surface peripheral electrical stimulation on nerve regeneration after digital nerve neurorrhaphy: study protocol for a randomized clinical trial.

https://doi.org/10.6084/m9.figshare.13584764.v1 ( de Santana Ribeiro de Mattos *et al.*, 2021d)

Data are available under the terms of the Creative Commons Zero “No rights reserved” data waiver (CC0 1.0 Public domain dedication).
