# Supplementary material for: Influence of surface peripheral electrical stimulation on nerve regeneration after digital nerve neurorrhaphy: study protocol for a randomized clinical trial
Source: F1000Res. 2021 Dec 6;10:219. Originally published 2021 Mar 17. [Version 2] doi: 10.12688/f1000research.42120.2 (PMC8596177; doi:10.12688/f1000research.42120.2)
Supplement: Peer review history

# Peer review: Reviewer response for version 1

**DOI:** 10.5256/f1000research.45186.r98628

**Published:** 2021-11-16

**Authors:** Jonas Kolbenschlag, Johannes Heinzel

**Affiliations:** 1 BG Trauma Center Tuebingen, Department of Hand, Plastic, Reconstructive and Burn Surgery, Eberhard Karls University Tuebingen, Tübingen, Germany; 2 BG Trauma Center Tuebingen, Department of Hand, Plastic, Reconstructive and Burn Surgery, Eberhard Karls University Tuebingen, Tuebingen, Germany

I think this is a highly interesting study protocol and the planned study has been thoughtfully conceptualized. I have no major concerns to raise, but I wonder whether the authors plan to include patients undergoing direct repair of non-segmental nerve defects only? I think this is implicated by using the term neurorrhaphy in the title of the manuscript but is not clearly stated in the protocol itself. Although I would deem an investigation of the effects of PES very interesting, I would recommend only to include patients with direct repair of non-segmental lesions.  An experimental and control(SHAM) group, respectively, with segmental nerve damages could be included in further experiments however, to study the effects of PES following reconstruction of segmental nerve lesions.

 All in all, I consider the work at hand highly interesting and a valuable addition to the field.

Is the study design appropriate for the research question?

Yes

Is the rationale for, and objectives of, the study clearly described?

Yes

Are sufficient details of the methods provided to allow replication by others?

Yes

Are the datasets clearly presented in a useable and accessible format?

Yes

Reviewer Expertise:

Peripheral Nerve, Microcirculation

We confirm that we have read this submission and believe that we have an appropriate level of expertise to confirm that it is of an acceptable scientific standard.

# Response

**Published:** 2021-11-26

**Authors:** ENILTON MATTOS

**Affiliations:** HUPES, Brazil

Thank you for the good comment. We agree with your comment that the repair of non-segmental nerve defects is not clearly stated in the protocol and we have corrected it.

 Best regards,

 Enilton Mattos

# Peer review: Reviewer response for version 1

**DOI:** 10.5256/f1000research.45186.r86882

**Published:** 2021-06-22

**Authors:** Yumin Yang

**Affiliations:** 1 Key Laboratory of Neuroregeneration of Jiangsu and Ministry of Education, Co-innovation Center of Neuroregeneration, Nantong University, Nantong, China

I think this is an excellent manuscript, and it deserves to be accepted. This article studied the influence of low intensity and frequency surface peripheral electrical stimulation (PES) on nerve regeneration of digital nerve injuries of the hand after its surgical repair in humans. This study could provide novel, useful, and practical strategies to treat patients with peripheral nerve damage. If the effects of different stimulus intensity, time and stimulus parts can be studied more systematically, it will be a more perfect study.

Is the study design appropriate for the research question?

Yes

Is the rationale for, and objectives of, the study clearly described?

Yes

Are sufficient details of the methods provided to allow replication by others?

Yes

Are the datasets clearly presented in a useable and accessible format?

Yes

Reviewer Expertise:

Influence of surface peripheral electrical stimulation on nerve regeneration

I confirm that I have read this submission and believe that I have an appropriate level of expertise to confirm that it is of an acceptable scientific standard.

# Response

**Published:** 2021-06-23

**Authors:** ENILTON MATTOS

**Affiliations:** HUPES, Brazil

Dear professsor Yumin Yang,

 Thank you very much for the considerations.

 Best regards,

 Enilton Mattos
